# Reliability of pelvic parameters measurement

**DOI:** 10.1186/1748-7161-8-S2-P3

**Published:** 2013-09-18

**Authors:** Julie Deceuninck, Jean-Claude Bernard, Eric Berthonnaud

**Affiliations:** 1 Croix Rouge française - CMCR des Massues, Lyon, France

## Background

Pelvic parameters are an essential measurement in sagittal radiographic analysis. However, it is difficult to control the patient position during radiograph and it is possible that a strict profile cannot be obtained. 

## Method

Fourteen standing biplanar radiographic files of asymptomatic and scoliotic patients have been recorded and treated in the frame of pelvis/spine studies. Radiographic examinations involved frontal and sagittal exposures grasped successively. A standard radiographic set up is used, involving a rotating platform, interposed between radiographic source and plate. Patients must stand motionless on the platform, with bearing poles helping patients to keep a stable posture. Two numerical radiographs (size 30 cm x 90 cm) are shot. A self-calibration procedure is then applied to the two radiographs, which takes into account small patient movements occurring between successive grasps. The self-calibration technique is based upon epipolar plane geometric properties. Pelvic parameters—pelvic tilting and pelvic incidence—are measured clinically on sagittal x-ray. Direct measurements on sagittal x-ray of pelvic tilting and incidence are not accurate when the standing patient’s pelvis is tilted while radiographed. Angular components calculated from a 3-dimensional analysis of pelvis shape and orientations are compared with corresponding values measured on sagittal x-ray. 

## Results

Fourteen examples of standing pelvis are presented. Pelvic tilting and incidence angles are obtained from 2-dimensional measurements and 3-dimensional analysis. In some cases, corresponding angular values are close together. In other examples, results differ significantly. 

## Conclusion and discussion

The single sagittal radiograph of the pelvis cannot explain such differences, contrary to results extracted from 3-dimensional analysis. Therefore, we must be careful in the reading of our pelvic parameters measurements. 
